# Vitamin D and COVID-19: An Update on Evidence and Potential Therapeutic Implications

**DOI:** 10.7759/cureus.46121

**Published:** 2023-09-28

**Authors:** Aakanksha V Tiwari, Suwarna Dangore-Khasbage

**Affiliations:** 1 Oral Medicine and Radiology, Sharad Pawar Dental College and Hospital, Datta Meghe Institute of Higher Education and Research, Wardha, IND; 2 Oral Medicine and Radiology, Sharad Pawar Dental College and Hospital, Datta Meghe Institute of Medical Sciences, Wardha, IND

**Keywords:** post covid complications, immunity, vitamin d, sars-cov-2, covid-19

## Abstract

The COVID-19 pandemic created havoc in the whole world since 2019. It is an explosively spreading infectious disease in which the infectious agent enters the body through sneezing coughing touching etc. The primary site of infection is the respiratory system, and the various common symptoms are dry cough, fever, dyspnea, sore throat, nasal congestion, and loss of taste sensation. A majority of the patients experience diarrhea, vomiting, severe headache, and muscle pain. Many research have been undertaken to study the therapeutic implications of different elements in coronavirus infection. One such element of interest is vitamin D. There is evidence in the literature regarding the usefulness of vitamin D in severe acute respiratory distress syndrome (ARDS) and several respiratory diseases. As the site of infection in coronavirus infection is primarily the respiratory system, reviewing in detail the correlation of this vitamin with SARS-CoV-2 infection, is an area of keen interest. Thus, the aim of this article is to explore and describe in detail the relation between the two, with reference to levels of this vitamin in diagnosed subjects and a need for its supplementation in the management of coronavirus infection and also in the prevention of post-COVID-19 complications. The review concluded that Vitamin D has an immunomodulating function. Its deficiency may lead to severe respiratory illnesses including ARDS. Vitamin D levels affect the disease course in COVID-19 infection and proper blood concentration can reduce the severity of the symptoms as well as post-COVID-19 complications.

## Introduction and background

The world has experienced a major pandemic of coronavirus infection. It was initiated in Wuhan, Hubei, China, from where it explosively spread to the whole world. World Health Organization on February 11, 2019 named the outbreak as COVID-19 [1]. It is a contagious respiratory disease caused by the beta coronavirus virus, which causes SARS-CoV-2 infection [2].

Scientists worldwide are engaged in numerous types of research on various elements, drugs, herbal medications, vitamins, etc. to find out the best possible approach to curtail the symptoms and bring down its severity in active infection, curtail the post-infection complications, and add to finding out the therapeutic implication of such element in treating the active cases.

Vitamin D is one such element of interest. Vitamin D is a secosteroid that is soluble in adipose tissue and is involved in calcium and phosphate metabolism in the body. It is moreover considered a hormone rather than a vitamin [3,4]. It is an essential vitamin that can be synthesized in the skin (cholecalciferol) or can be consumed from meals (ergocalciferol). Sunlight is the primary source for the synthesis of this vitamin. It is commonly known as “sunlight hormone” or “sunshine vitamin” [5]. The transformation of 7-dehydrocholesterol available in the skin (inactive vitamin) to 1,25-dihydroxyvitamin D3 (active Vitamin) is carried out through multiple series of events in the liver and kidney [6]. This series of events depends on hormones in the liver and kidney for the formation of vitamins that can function actively in the body. Pathological conditions of the liver and kidneys may cause insufficient production of the active form of the vitamin leading to its deficiency. It plays various other important roles other than calcium and phosphate metabolism such as in reducing inflammation through innate and adaptive immunity, maintaining the tonicity of cardiac muscles, and preventing lung injury [7].

Numerous studies are being performed on this Vitamin to test its therapeutic implications in COVID-19 infection. The purpose of studying the action of this Vitamin in COVID-19 infection is because of the available evidence from literature about its effect in severe acute respiratory distress syndrome (ARDS), and many other respiratory diseases. This disease primarily involves the respiratory system attracting attention to study and correlate the effectiveness of vitamin D in subjects infected with coronavirus infection. This article reviews the action of this vitamin in reducing the severity of infection in infected subjects and its effectiveness in preventing post-COVID-19 complications.

## Review

Role of vitamin D3 in host immune response

**Figure 1:**
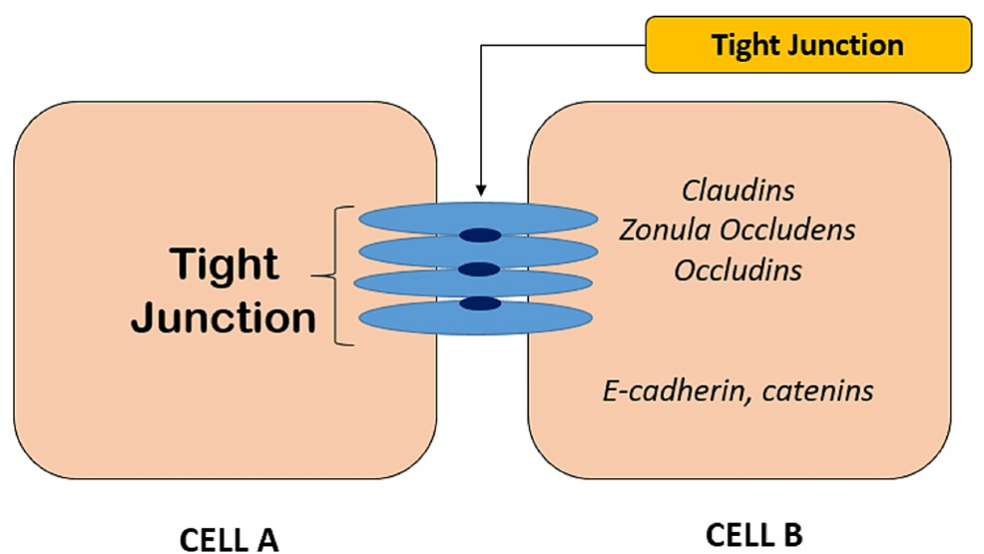
Tight junction and adherens junction between the epithelial cells acting as physical barriers to the entry of infectious pathogens Claudins, Zona Occludens, Occludins, E-cadherins, Catenins - Transmembrane proteins which bind one cell to another cell

This Vitamin has a crucial role in immune-modulating actions, i.e., it influences both innate and adaptive immunity [8,9]. In innate immunity, it maintains the integrity of the physical barriers, i.e., the tight junction and adherens junction (Figure 1) between the cells which combat the entry of infectious pathogens within the body.

**Figure 2:**
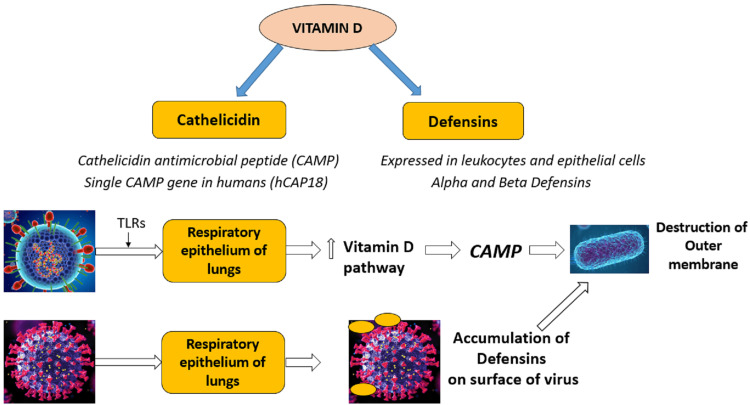
Role of vitamin D through cathelicidin and defensin immune proteins in reducing viral load in respiratory infection CAMP - Cathelicidin antimicrobial peptide - Host defence peptides with antimicrobial and immunemodulatory function, hCAP18 - Human cationic antimicrobial protein 18 - Single cathelicidin antimicrobial peptide gene in human body characterized by conserved cathelin proregion and variable antibacterial peptide in C-terminal domain, TLRs - Toll like receptors - Important mediators of inflammatory pathways which play major role in mediating immune responses and it links adaptive and innate immunity

It has an effect on adaptive immunity. It increases the secretion of Human Cathelicidin LL-37 and Defensin immune proteins [8]. When respiratory syncytial virus (RSV) enters the lung alveoli through respiration, andreacts with respiratory epithelium through TLR3 (toll-like receptors 3) which forms the group of vital mediators of the inflammatory cascade in the gut that perform a crucial role in evoking the response of the immune system toward different types of pathogen-derived ligands and establish the correlation of adaptive immunity with the innate immunity, there is an upregulation of Vitamin D metabolism pathway, which in turn leads to the formation of cathelicidin antimicrobial polypeptide (CAMP) [10-12]. There is a single (CAMP) gene in the human body, i.e., hCAP18 [12]. This protein destroys the outer membrane of the pathogen thereby causing the destruction of the pathogen ultimately reducing the viral load (Figure 2).

Defensins are secreted in leukocytes and epithelial cells. They are of two types the Alpha and the Beta defensins. They act on the influenza virus. As the virus enters the respiratory system, defensins get attached to the influenza virus and accumulated onto the surface of the virus [10,11]. This reduces the virulence of the virus. Ultimately leading to the destruction of the outer cell membrane thus reducing the viral load (Figure 2). Both these immune proteins also function for chemo-attraction of cells of the immune system to the site of inflammation via chemotaxis thereby reducing inflammatory response.

Different vital functions of sunlight hormone

Sunlight hormone block the Angiopoietin Ang-2-Tie-2 signaling pathway. Angiopoietin comes as a part of a group of vascular growth factors that function in embryonic and postnatal angiogenesis [13]. The angiopoietin (Ang)-Tie signaling pathway is a crucial signaling pathway that carries out the development of vascular apparatus, formation of newer blood vessels, and remodeling, as well as in the regulation of permeability of blood vessels, homeostasis, quiescence, and stability. In inflammatory conditions, there is an activation of this pathway leading to an increased vascular permeability. Vitamin D blocks this pathway and reduces the symptoms of inflammation [14]. Another important function is it acts as a negative endocrine Renin Angiotensin System (RAS) modulator. RAS has a significant action in maintaining the physiology of renal, cardiac, and vascular systems and as it is centrally activated, many common pathologic conditions like high blood pressure, acute heart failure, and renal diseases pose a great risk. It has a negative modulator effect on this system thus preventing the abovementioned sequel [15]. Vitamin D suppresses the proinflammatory cytokines and reduces the cytokine storm, which is responsible for the symptoms in various inflammatory conditions. Specifically, IL-1β, IL-6, and TNF-α are responsible for inflammatory response. A decrease in the levels of these cytokines will reduce the symptoms of inflammation [13-15].

Outcomes of vitamin D3 deficiency on the human body

Vitamin D deficiency can be caused due to various reasons such as insufficient exposure to sunlight, insufficient dietary intake, and pathologies involving the liver and kidneys [16]. The levels of active vitamin below 20ng/mL (50nmol/L) and the in range 21-29ng/mL (52 to 72nmol/L) have been described as deficiency and insufficiency, respectively, by the Endocrine Society. Deficiency of the functioning form of this Vitamin has adverse effects on various organs of the body [16].

Vitamin D deficiency needs to be evaluated for subjects expected to be at higher risk as it is more common than ever. The correlation between deficiency of sunlight hormone and cancer, cardiovascular illness, diabetes, autoimmune diseases, and neuropsychiatric disorders has been the subject of numerous contradicting research in recent years [17]. Various countries across whole world revealed vitamin D deficiency. In India, and some other countries more than 20% of the subjects experienced vitamin D levels less than 30 nmol/L (or 12 ng/mL). Moreover, it was concluded that 490 million population in India was deficient in vitamin D [17]. There is a relatively high frequency of vitamin D insufficiency in some patient subgroups. They are frequently characterized by a deficiency in or impaired function of organs concerned with the metabolism of this vitamin. Between 85% and 99% of patients with chronic kidney disease who are undergoing dialysis, patients who are posted to receive renal transplants who have hepatic disease, or patients who have undergone liver transplantation may be deficient in vitamin D [17].

**Figure 3:**
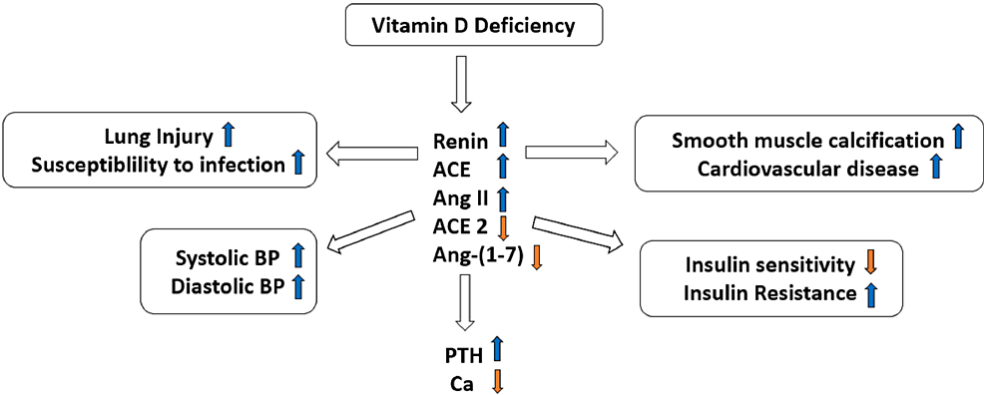
Effects of vitamin D deficiency on human body ACE - Angiotensin-converting enzyme - It is a central component of the renin angiotensin system, which controls blood pressure by regulating the volume of fluids in the body. It converts the hormone angiotensin I to the active vasoconstrictor angiotensin II, Ang II - Angiotensin II - Active form of vasoconstrictor, PTH - Parathyroid hormone - A hormone released by parathyroid glands to control calcium levels in your blood, Ca - Calcium, BP - Blood pressure

Ibrahim et al. suggested a case of a married, 26-year-old Sudanese woman with a three-year-old kid was the subject of this lawsuit. This patient came with the complaint of anosmia for the past two years. She also complained of generalized fatigue and easy tiredness even on performing daily simple activities [18]. She had lower back pain without any previous history of trauma or strenuous physical activity. With the exception of bilateral nasal polyps, all system examinations were normal. The laboratory findings showed a low vitamin D level of 7 ng/mL, which was consistent with a vitamin D insufficiency being suspected. She was instructed to take foods high in vitamin D and to have at least 20 minutes of exposure to the sun thrice a week following the loading dose of the supplement [18]. The recommended dosage for supplementation was 50,000 (IU) every week for eight weeks. During that time, it was seen that the signs of fatigue, sleepiness, anosmia, and back pain were significantly improving [18]. Figure 3 depicts the deleterious outcomes of its deficiency on the human body.

Relationship of ACE2 and COVID-19 virus

Viral infection begins with internalization into host cells. Particular receptors on the cell membrane of the host can bind to a spike protein present on the envelope of the virus. Previous research has demonstrated that the viral-specific receptor is (ACE 2) [16]. In order to prove that ACE2 is the COVID-19 cell receptor, Zhou et al. demonstrated that corona virus may get into cells that possess ACE2 receptors but cannot get inside the cells that lack it or cells those possess other viral receptors, such as aminopeptidase N and dipeptidyl peptidase 4 (DPP4) [19]. Further research revealed that the corona virus's spike protein's affection for ACE2 is 10 to 20 times more as compared to that of the other coronavirus receptors. After getting bonded together, there is fusion of viral and host cell membranes, which initiates infection by expelling viral RNA into the cytoplasm. Infected ACE2 receptor is internalized with the virus during infection [20]. The spike glycoprotein does not obstruct ACE2's catalytically active region, and the mechanism of bonding is not dependant of ACE2's peptidase activity. Several transmembrane protein cleaving enzymes and proteins, which includes vimentin and clathrin, might be implicated in the attaching process and various processes involving the membrane fusion [20]. As an example, these protein cleaving enzymes include serine 2, transmembrane protease serine 2(TMPRSS2), disintegrin and metallopeptidase domain 17 (ADAM17), and TNF-converting enzyme. For instance, TMPRSS2 and ADAM17 are known to be able to breakdown ACE2 so that the viral uptake is increased and an increased ectodomain shedding, respectively [20]. ACE2 closely represents ACE which is present on different organs of the body [21]. ACE2 to variable degrees are present over various human organs. The immunohistochemistry and the advanced single-cell RNA-sequence analysis of the respiratory system revealed that ACE2 is primarily present on cells of epithelium in alveoli of lungs which are type II cell, but only poorly expressed on the surface of oral epithelium and mucosal surface of nasal cavity, nasopharynx and trachea [21]. This revealed the impression the main site of COVID-19 infection is lungs. It functions to prevent acute injury to lungs in conditions of severe acute respiratory distress. Through blood circulation, the corona virus which are free of any cellular adhesion and those viruses which are associated with macrophage - phagocytosis, might migrate from the lungs to different sites of the body where ACE2 are abundantly expressed [21]. It functions to prevent acute injury to lungs in conditions of severe acute respiratory distress. ACE2 acts as a catalyst and brings about the conversion of angiotensin II to angiotensin-(1-7), and the ACE2/angiotensin-(1-7)/MAS axis reduces severe negative results of the renin-angiotensin system (RAS), that acts to maintain physiology and pathophysiology of the body [22]. Adding to the effects of the virus those are direct in nature and factors associated with inflammation and immuntiy that are responsible for COVID-19 pathogenesis, suppression of ACE2 and disturbed balance between the RAS and ACE2/angiotensin-(1-7)/MAS following the infection might also result in damage to multiple body organs in COVID-19 infection [22]. The spike protein of the virus, that attach to ACE2, is a strong target for evolving drugs specific to that particular protein, antibodies, and vaccines. Correcting the disturbed balance between the RAS and ACE2/angiotensin-(1-7)/MAS might significantly help to protect damage to the organs [22].

**Figure 4:**
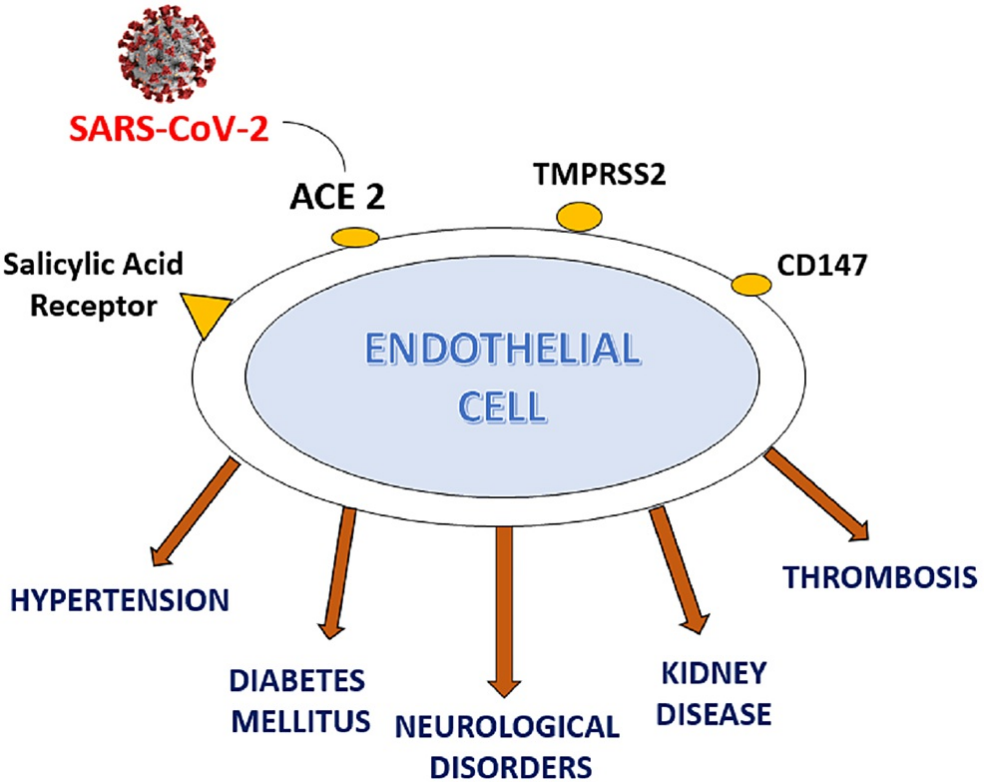
Angiotensin-converting enzyme-2 receptor present on the outer membrane of endothelial cell which depicts entry point of the virus into the cell and effect of severe COVID-19 infection in hospitalized patients SARS-CoV-2 - Severe acute respiratory syndrome coronavirus 2 - A strain of coronavirus that causes COVID-19, the respiratory illness responsible for the COVID-19 pandemic, ACE 2 - Angiotensin-converting enzyme 2 - Enzyme that converts inactive angiotensin I to active form angiotensin II, TMPRSS2 - Transmembrane protease serine 2 - It is involved in the cleaving peptide bonds of proteins that have serine as the nucleophilic amino acid within the active site, CD147 - Cluster of differentiation 147 - Regulates cell proliferation, apoptosis, and tumor cell migration, metastasis and differentiation, especially under hypoxic conditions. CD147 is also important to many organ systems

Symptoms of corona virus disease are mentioned earlier in the text. Along with the mentioned symptoms majority of patients experienced diarrhoea, vomiting, severe headache and muscle ache [23]. The alarming sign being severe deprivation in oxygen saturation poses an urge to immediately hospitalize the patient and provide external oxygen support. Figure 4 depicts the mechanism of entry of corona virus inside the endothelial cell through ACE 2 receptor and its effects in hospitalized patients.

Role of vitamin D3 in patients infected with corona virus

Viral infections are more common when vitamin D levels are low, although there are few precise data on COVID-19. It is known that 40% to 70% of people who are seriously ill have vitamin D insufficiency. In a study by Notz et al., 25-hydroxyvitamin D deficiency was present in 85% of individuals with fulminating respiratory illness. All subjects who did not have vitamin D in their prescription at home experienced levels below 30 ng/mL [20]. The evidence revealed a high occurrence of vitamin deficiency in infected patients who are critically ill. While studies indicate that insufficiency of sunlight hormone favors the formation of active form of vitamin D over 24,25-dihydroxyvitamin D to maintain homeostasis of calcium as and when possible, only a minority of people had deficiencies for 1,25-dihydroxyvitamin D [20].

**Figure 5:**
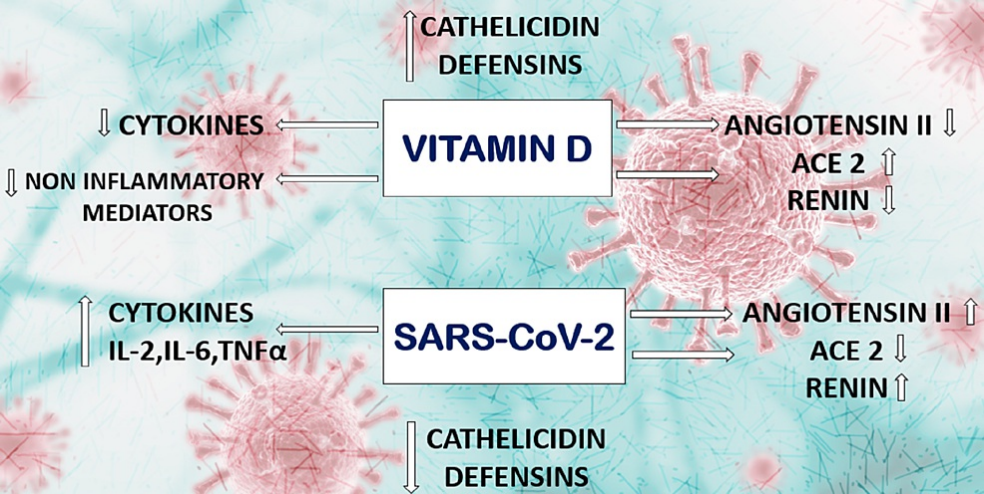
Effects of severe acute respiratory syndrome - coronavirus 2 and Vitamin D on various systems of the body SARS-CoV-2 - Severe acute respiratory syndrome coronavirus 2 - A strain of coronavirus that causes COVID-19, the respiratory illness responsible for the COVID-19 pandemic, IL-2, IL-6 - Interleukin 2, Interleukin 6 - A type of cytokine signaling molecule in the immune system, TNF - Tumor necrosis factor - Major regulator of inflammatory responses and is known to be involved in the pathogenesis of some inflammatory and autoimmune diseases, ACE 2 - Angiotensin-converting enzyme 2 - Enzyme that converts inactive angiotensin I to active form angiotensin II

As discussed earlier this vitamin plays a significant role in corona virus infection by down-regulating the Ang-2-Tie-2 signaling pathway, by having a negative modulatory action of endocrine RAS, suppression of cytokine storm etc. Figure 5 summarizes the usefulness of this vitamin in infected patients.

Vitamin D in post COVID-19 complications

Vitamin D3 is an effective antioxidant and anti-inflammatory agent. It is recognized that toxins, long-term metabolic disorders, and aging contribute to mitochondrial malfunction. Abnormal mitochondria produce insufficient ATP while producing excessive reactive oxygen species (ROS), intensifying and prolonging the effects of excessive oxidative stress. These occurrences result in accelerated aging, early cell death, and DNA damage (and compromise DNA repair mechanisms). Data are accumulating that suggest prolonged intracellular inflammation, as is the situation with vitamin D deficiency, is likely what fuels mitochondrial dysfunction [24]. One of vitamin D's main roles is to control oxidative stress, chronic inflammation, and maintain mitochondrial respiratory processes. Vitamin D has important positive impacts on regulating oxidative stress, inflammation, and energy metabolism through its focused mitochondrial activity and subduing of ROS through a variety of pathways [24]. Having enough of it considerably reduces the risk of developing illnesses where the pathognomonic feature of the development and progression of illness is an inflammatory process. Such illnesses for example include rheumatoid arthritis, illness concerned with short-term memory loss, multiple sclerosis, and cancer [25]. The fibrotic and “cytokine storm” effects of infection can be countered by vitamin D3. The proinflammatory transcription factor nuclear factor kappa light-chain enhancer of activated B cells (NFkB) is inhibited by the NFkB gene enhancer in B-cells inhibitor alpha (IkB), which decreases the expression of inflammatory genes [25]. This vitamin can influence the genetic presentation of the interleukins IL-1, IL-2, IL-4, IL-6, IL-8, IL-10, and IL-15, interferon gamma (IFN-), tumor growth factor beta (TGF-), and tumour necrosis factor alpha (TNF-), according to dose and cell-specific mechanisms, in ways that support tissue repair and anti-inflammatory pathways [26]. The VDR receptor is present in T cells which are activated and are matured [26]. The proinflammatory Type-1 T helper (Th1) and Type-17 T helper (Th17) subtypes which induce injury to the tissues are suppressed by the immunomodulatory effect of this vitamin on CD4+ T-cells in favor for activation of Type-2 T helper (Th2) cells and induced regulatory T-cells (iTreg). Through dose-dependent TGF- induction, this shift towards Th2 and iTreg subtype supports B-cell activation in humoral immunity, effects combating the inflammation, and pathways for repair of the damaged tissues. Studies have not demonstrated that vitamin D3 encourages the proliferation of T cells from naive precursors [26]. Sulli et al. demonstrated that serum insufficiency of vitamin D was linked to severe and extensive pulmonary involvement, long course of the disease, and risk of death in a group of geriatric patients aged 70 hospitalized for infection [27]. These senior participants had considerably lower 25(OH)D3 levels than the controls. D-Dimer and C-reactive proteins, two crucial markers of coagulopathy and inflammation, were substantially linked with higher 25(OH)D3 blood concentrations [27].

**Figure 6:**
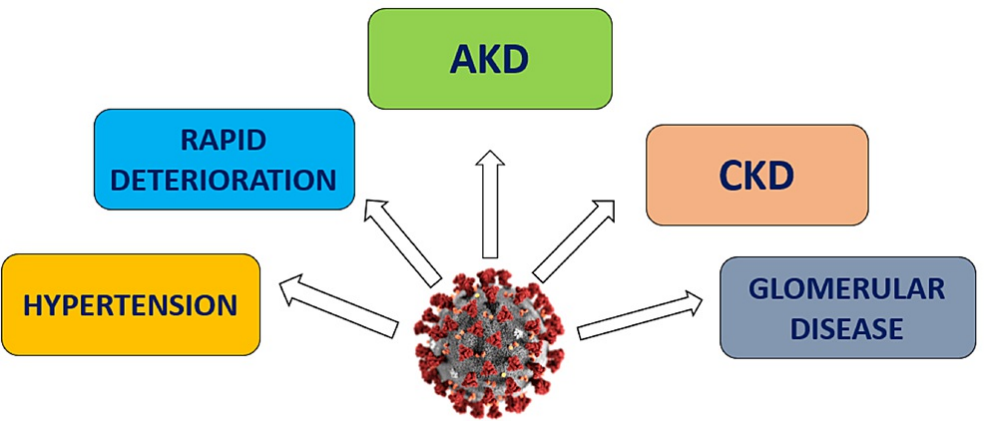
Post-COVID-19 complications leading to damage to various organs of the body AKD - Acute kidney disease - A condition in which the kidneys suddenly cannot filter waste from the blood, CKD - Chronic kidney disease - A gradual loss of kidney function over time

Post COVID-19 complications are mainly due to various comorbidities associated with the infection. Conditions affecting the cardiovascular system (most common being the hypertension), musculoskeletal, pulmonary, hematological, renal, endocrine, gastrointestinal and neuropsychiatric systems are found to be significantly associated with the infection [28]. Vitamin D has a negative modulating effect for all these comorbidities through its action on various systems as already explained in the text previously, thereby reducing the risk of severe complications following the infection [28]. Diabetes mellitus being the most dangerous, when combined with steroid treatment can lead to serious complications like mucormycosis. Figure 6 shows the multiple post-COVID-19 complications where vitamin D has a protective role.

Vitamin D has negative modulating effects on all the abovementioned comorbid conditions, thereby reducing the risk of developing serious complications [29]. Improved health outcomes may be expected when vitamin D3 is administered. The benefits of vitamin D3 cholecalciferol supplementation on enhancing survival outcomes in elderly hospitalized patients were compared in an Italian quasi-experimental trial. Three groups of participants were created. The first group received vitamin D for a year prior to contraction of COVID-19 and hospitalization for the same. The second group was given an oral supplement of 80,000 IU of vitamin D3 after some time of the disease diagnosis, and the third group was reference Group 3 which received no supplements [29]. According to these results, regular bolus vitamin D3 intake as a lifestyle choice or preventative habit prior to virus infection was linked to a better prognosis and increased survival in frail elderly people. This result combines evidence from earlier research suggesting those who have enough levels of 25(OH)D3 in their circulation through proper amount of vitamin D intake or adequate exposure to sunlight in the past show a significantly high rate of survival as compared to ones with its deficiency [29]. It suggests that maintenance of proper levels of this vitamin in people prior to disease might be of preventative utility and improve public health. In the future, large population studies should be carried out to confirm supplementing requirements, dosages, and regimens, as well as to explore long-term vitamin D3 administration [29].

Controversies in the field

There is little debate that vitamin D levels strongly correlate with a variety of COVID-related outcomes in observational studies (e.g., retrospective, cohort, etc.), that true deficiency is likely to worsen infectious outcomes, including those from COVID, and that vitamin D is essential for a variety of normal immunological functions. Why, therefore, save maybe in individuals with the absolute lowest levels, do all the randomized studies conducted yet fall short of the lofty expectations that vitamin D treatment will prevent or alleviate COVID-19 [30]? According to what I understand, a deficiency state happens when a substance is deficient, and this deficiency results in a pathological condition that can be treated or avoided by replacing or supplementing the missing substance. Numerous nutrients frequently have low blood levels due to disease activity, although these low levels do not actually indicate shortages. Take calcium as an illustration. Albumin carries the majority of the calcium in the blood. Every doctor is instructed to modify the calcium interpretation based on albumin levels. There is no deficient state when there is a low calcium level, low albumin level, and systemic inflammation. Patients with low vitamin D levels should not be deemed to have a deficiency if supplementation is offered to them and results are not improved [30]. In our eagerness to show how important nutrition is, we frequently overlook this kind of connection between nutrients and the severity of a disease. There are, in my opinion, two really vital reasons why this needs to be explored. First, it is unnecessary to continue spending money on observational studies that link vitamin D to COVID-19 outcomes. Second, when scientists and other experts fail to keep an eye on how research findings are communicated to science consumers, the credibility of science continues to suffer. The public's trust in science is compromised when benefits are extolled or even theorized before being disproven [30].

## Conclusions

Vitamin D has a key role management of respiratory infection, and it is briefly explained in the article. Considering respiratory system as the primary site to be affected by COVID-19 infection, the estimation of the vitamin D levels can be considered one of the important investigations in this infection, especially in cases of critically ill and hospitalized COVID patients. Similarly taking into consideration the multiple beneficial roles of Vitamin D on the human body, the supplementation of Vitamin D to COVID-19-positive individuals in an attempt to control the severity of infection as well as to prevent post-COVID-19 complications is of vital significance. Overall, the immune system is immunomodulated by vitamin D. Its effects on the likelihood of contracting the illness and its progression are thoroughly explained in the literature. The importance of this vitamin in treating COVID-19 cannot currently be advised, but a less complicated course of the disease is unquestionably favored by its proper blood concentration value. The blood vitamin D content is affected by pandemic-related constraints, primarily isolation and quarantine, and may therefore call for supplementation. Further research is needed to evaluate whether vitamin D could be considered a treatment option for COVID-19-infected subjects. To conclude with the currently available evidence, evaluation of Vitamin D values in COVID-19 patients should be considered, and adequate supplementation of the same should be undertaken to benefit the affected individuals and reduce the risk of complications.
